# Parathyroid Hormone (PTH)–Induced Bone Gain Is Blunted in *SOST* Overexpressing and Deficient Mice

**DOI:** 10.1359/jbmr.090730

**Published:** 2009-07-13

**Authors:** Ina Kramer, Gabriela G Loots, Anne Studer, Hansjoerg Keller, Michaela Kneissel

**Affiliations:** 1 Musculoskeletal Disease Area, Novartis Institutes for BioMedical Research CH-4002 Basel, Switzerland; 2 Biology and Biotechnology Division, Lawrence Livermore National Laboratory 7000 East Avenue, L-452 Livermore, CA, USA; 3 Department of Molecular and Cell Biology, Division of Genetics, Genomics, and Development, and Center for Integrative Genomics, University of California–Berkeley Berkeley, CA, USA

**Keywords:** PTH, Sost, osteocytes, genetic mouse models, osteoporosis

## Abstract

Intermittent parathyroid hormone (PTH) treatment is a potent bone anabolic principle that suppresses expression of the bone formation inhibitor *Sost*. We addressed the relevance of *Sost* suppression for PTH-induced bone anabolism in vivo using mice with altered *Sost* gene dosage. Six-month-old *Sost* overexpressing and 2-month-old *Sost* deficient male mice and their wild-type littermates were subjected to daily injections of 100 µg/kg PTH(1–34) or vehicle for a 2-month period. A follow-up study was performed in *Sost* deficient mice using 40 and 80 µg/kg PTH(1–34). Animals were sacrificed 4 hours after the final PTH administration and *Sost* expression in long bone diaphyses was determined by qPCR. Bone changes were analyzed in vivo in the distal femur metaphysis by pQCT and ex vivo in the tibia and lumbar spine by DXA. Detailed ex vivo analyses of the femur were performed by pQCT, µCT, and histomorphometry. Overexpression of *Sost* resulted in osteopenia and *Sost* deletion in high bone mass. As shown before, PTH suppressed *Sost* in wild-type mice. PTH treatment induced substantial increases in bone mineral density, content, and cortical thickness and in aging wild-type mice also led to cancellous bone gain owing to amplified bone formation rates. PTH-induced bone gain was blunted at all doses and skeletal sites in *Sost* overexpressing and deficient mice owing to attenuated bone formation rates, whereas bone resorption was not different from that in PTH-treated wild-type controls. These data suggest that suppression of the bone formation inhibitor *Sost* by intermittent PTH treatment contributes to PTH bone anabolism. © 2010 American Society for Bone and Mineral Research

## Introduction

Osteoporosis is a systemic skeletal disorder characterized by low bone mass and microarchitectural deterioration of bone tissue with a consequent increase in bone fragility and susceptibility to fracture.(1) It is characterized by a disturbed remodeling balance of bone resorbing osteoclasts and bone forming osteoblasts, resulting in a net decrease in bone mineral density (BMD). Most osteoporosis therapies are based on inhibition of bone catabolic osteoclast activity and hence can merely prevent further bone loss. The only clinically approved bone anabolic therapy, which can restore bone, is intermittent parathyroid hormone (PTH) treatment by daily injection.(2,3) While it is an established bone forming principle, the underlying molecular mechanisms are only partially understood. PTH affects cells of the mesenchymal osteoblast lineage by signaling through the G-protein-coupled type 1 PTH/PTH-related peptide receptor (PTH1R).(4) It has been shown to increase osteoblast number by promoting osteoblastic precursor proliferation(5,6) and reactivating quiescent bone lining cells,(7) as well as by stimulating osteoblast survival.(8,9) In addition, intermittent PTH treatment enhances osteoblast differentiation and activity.(10) Finally, emerging evidence suggests a putative role for osteocytes in PTH-induced bone anabolism.(11–13)

Osteocytes represent the majority of all bone cells. They are terminally differentiated cells of the osteoblast lineage embedded within the mineralized bone matrix.(14) They are interconnected with each other and with osteoblasts and lining cells on the bone surface via dendritic processes, forming a communication network throughout the bone matrix and to the bone surface that seems ideally suited for sensing and responding to the needs of the skeleton. Osteocytes express not only *PTH1R*(15,16) but also express *Sost*, a potent bone-formation inhibitor,(17,18) suggesting that they might play an important function in control of bone homeostasis. We and others recently found that PTH signaling suppresses the expression of *Sost* at the transcriptional level in a direct manner.(11,12) Consistent with *Sost* encoding sclerostin, a secreted glycoprotein inhibiting bone formation, transgenic mice overexpressing human *SOST* exhibit a low bone mass phenotype.(19,20) Conversely, constitutive *Sost* knockout (KO) mice lacking *Sost* expression display a high bone mass phenotype and increased bone strength.(21) Moreover, patients afflicted by sclerosteosis (MIM 269500)(22,23) or van Buchem disease (MIM 239100)(24,25) display lifelong bone overgrowth and a high bone mass phenotype with strongly increased bone mineral mass, density, and strength owing to loss of *SOST* expression.

The role of PTH signaling in osteocytes for the regulation of bone homeostasis was addressed recently by overexpressing a constitutively active PTH1R variant selectively in osteocytes.(13) Constitutively active PTH1R transgenic mice display increased bone mass and turnover and a concomitant reduction in *Sost* expression. These findings, together with the earlier observation that *Sost* is suppressed by PTH signaling,(11,12) suggest that osteocytes might contribute to PTH-induced bone anabolism and that suppression of *Sost* may play a role in the bone anabolic activity of intermittent PTH treatment.

To address the relevance of *Sost* suppression for PTH-induced bone gain, we employed genetically engineered mouse models with altered *Sost* expression levels. We subjected *SOST* transgenic (*SOST Tg*) mice overexpressing human *SOST* and homozygous *Sost* KO mice lacking endogenous mouse *Sost* to 2 months of intermittent bone anabolic PTH treatment. We hypothesized that the contribution of suppression of *Sost* for PTH-induced bone anabolism might be reduced under conditions of increased *Sost* gene dosage. Likewise, we reasoned that if PTH-induced suppression of *Sost* contributes to PTH-dependent bone anabolism in vivo, the bone anabolic response induced by intermittent PTH treatment should be attenuated in homozygous *Sost* KO mice, which lack *Sost* constitutively. Alternatively, if transient suppression of *Sost* is dispensable for PTH-induced bone anabolism in vivo, we expected to detect additive effects of *Sost* deficiency and intermittent PTH treatment. Here we report that PTH-induced bone anabolism is blunted in *Sost* overexpressing and deficient mice, suggesting that suppression of *Sost* contributes to PTH-induced bone gain in vivo.

## Materials and Methods

### Mice

The generation of *SOST* bacterial artificial chromosome (BAC) transgenic (*SOST Tg*) mice was described previously.(20) *SOST Tg* mice were maintained on a mixed FVB-C57BL/6 background. *Sost* KO mice with a targeted disruption of the *Sost* coding region were licensed from Deltagen, Inc. (San Mateo, CA, USA). Briefly, targeted ES cells derived from the 129/OlaHsd mouse substrain were used to generate chimeric mice, which were bred with C57BL/6 mice. Heterozygous *Sost* KO offspring were backcrossed for 4 generations to C57BL/6 mice and then interbred to generate homozygous *Sost* mutant mice. All mice were kept in cages under standard laboratory conditions with constant temperature of 25°C and a 12-12-hour light-dark cycle. Mice were fed on a standard rodent diet (3302, Provimi Kliba SA, Switzerland) with water ad libitum. Protocols, handling, and care of the mice conformed to the Swiss federal law for animal protection under the control of the Basel-Stadt Cantonal Veterinary Office, Switzerland.

### Systemic intermittent PTH(1–34) treatment

Six-month-old *SOST Tg* and 1.5-month-old [or 2-month-old in case of the hPTH(1–34) dosing experiment] *Sost* KO male mice and their wild-type male littermates were assigned to treatment groups (*n* = 17 to 18 per group for the *SOST Tg* experiment, *n* = 5 to 10 per group for *Sost* KO experiments) according to cross-sectional total BMD in the proximal tibia metaphysis as measured by peripheral quantitative computed tomography (pQCT) as first rank and body weight as second rank such that comparable group means and standard variations between groups designated to receive vehicle or PTH treatment were achieved at baseline in order to minimize experimental artifacts. All animals received daily subcutaneous injections on 6 (for the *SOST Tg* experiment) or 5 days per week (for *Sost* KO experiments) of vehicle (0.1% bovine serum albumin in phosphate-buffered saline, pH 7.4; Invitrogen, Karlsruhe, Germany) or 100 µg/kg human PTH(1–34) peptide [hPTH(1–34); Bachem, Bubendorf, Switzerland] with the exception of the hPTH(1–34) dosing experiment, in which treatment groups received 40 or 80 µg/kg hPTH(1–34). After 9 weeks of treatment, mice were sacrificed 4 hours after the final PTH injection by isoflurane or CO_2_-air inhalation followed by exsanguination.

### RNA extraction and quantitative real time PCR (qPCR) expression analyses

Total RNA was isolated from cortical bone of tibial or femoral diaphyses using TRIzol (Invitrogen, Karlsruhe, Germany) and RNeasy Mini (Qiagen, Hilden, Germany), as described previously,(11) and reverse-transcribed into cDNA using the High Capacity cDNA Reverse Transcription Kit (Applied Biosystems, Rotkreuz, Switzerland) according to the manufacturer's recommendations. Gene expression analyses were performed with an ABI Prism 7900HT sequence detection system (Applied Biosystems) using TaqMan Universal PCR Master Mix mouse or human glyceraldehyde-3-phosphate dehydrogenase (GAPDH) TaqMan assay reagents for normalization and TaqMan probes for mouse and human *Sost*/*SOST* according to the manufacturer's instructions (Applied Biosystems).

### Ex vivo dual-energy X-ray absorptiometry (DXA) analyses

The left tibia and lumbar vertebrae L1–4 were dissected, fixed for 24 hours in 4% phosphate-buffered paraformaldehyde, and stored in 70% ethanol. Tibial and lumbar spine BMD was measured in 70% ethanol for soft tissue calibration with a Hologic QDR-1000 instrument. A collimator with 0.9-cm-diameter aperture and an ultrahigh-resolution mode (line spacing 0.254 mm, resolution 0.127 mm) were employed.

### Peripheral quantitative computed tomographic (pQCT) analyses

Cross-sectional BMD, bone mineral content (BMC), and cortical thickness were evaluated in the left proximal tibia and distal femur metaphysis using an adapted Stratec-Norland XCT-2000 fitted with an Oxford (Oxford, UK) 50 µm X-ray tube (GTA6505M/LA) and a collimator of 0.5 mm diameter. The following setup was chosen for the measurements: voxel size 0.07 mm, scan speed scout view 10 mm/s, final scan 5 mm/s, 1 block, contour mode 1, peel mode 2, and cortical and inner threshold 350 mg/cm^3^. For in vivo measurements, animals were placed in the lateral position under inhalation narcosis (isoflurane 2.5%), and the left leg was stretched and fixed. For ex vivo analyses of the femur, six consecutive slices spaced equally along the entire femoral length were measured, and slices 1 to 5 were evaluated, with slice 1 being the most distal corresponding to the distal femur metaphysis.

### Micro-computed tomographic (µCT) analyses

Cancellous bone structure was evaluated ex vivo in the distal femur metaphysis using a Scanco vivaCT40 (voxel size 10.5 µm, high resolution, 50 slices, threshold 275; Scanco Medical AG, Bruttisellen, Switzerland).

### Tissue processing and bone histomorphometric analyses

All mice received fluorochrome markers by subcutaneous injection 10 days (alizarin complexone, 20 mg/kg; Merck, Zug, Switzerland) and 3 days prior to necropsy (calcein, 30 mg/kg; Fluka, Buchs, Switzerland) to evaluate bone formation dynamics. The left femur was fixed for 24 hours in 4% phosphate-buffered paraformaldehyde, dehydrated and defattened at 4°C, and embedded in methyl methacrylate resin. Per animal, a set of 5 µm nonconsecutive longitudinal sections was cut in the frontal midbody plane (Leica RM2155 microtome, Leica Microsystems, Heerbrugg, Switzerland). Fluorochrome-marker-based dynamic bone parameters were determined using a Leica DM microscope fitted with a Sony DXC-950P camera and adapted Quantimet 600 software (Leica Microsystems). Microscopic images of the specimen were digitized and evaluated semiautomatically on screen (200 × magnification) as described previously.(26) Bone surface, single- and double-labeled bone surface, and interlabel width were measured in the cancellous bone compartment of the distal femur metaphysis. Mineralizing surface [MS/BS = (dLS + sLS/2)/BS (%)] and mineral apposition rate [MAR (µm/day), corrected for section obliquity] were calculated, and the daily bone formation rate [BFR/BS (µm/d)] was derived. Osteoclast number was determined on 5 µm femoral microtome sections stained for tartrate-resistant acid phosphatase (TRAP) activity. Bone histomorphometric nomenclature was applied as recommended by Parfitt and colleagues.(27)

### Statistical analysis

All data shown represent mean ± standard error of the mean (SEM). Statistical analyses were performed using Student's *t* tests (two-tailed) or one-way analysis of variance (ANOVA). The level of significance is designated as follows: *^,X^*p* < .05 and **^,XX^*p* < .01. Unpaired Student's *t* tests were used for PTH- versus vehicle-treated mice of the same genotype (*, **) or for transgenic versus wild-type mice of the same treatment group (^X^, ^XX^).

## Results

### Skeletal parameters of *SOST Tg* mice and *SOST* expression

**Fig. 1:**
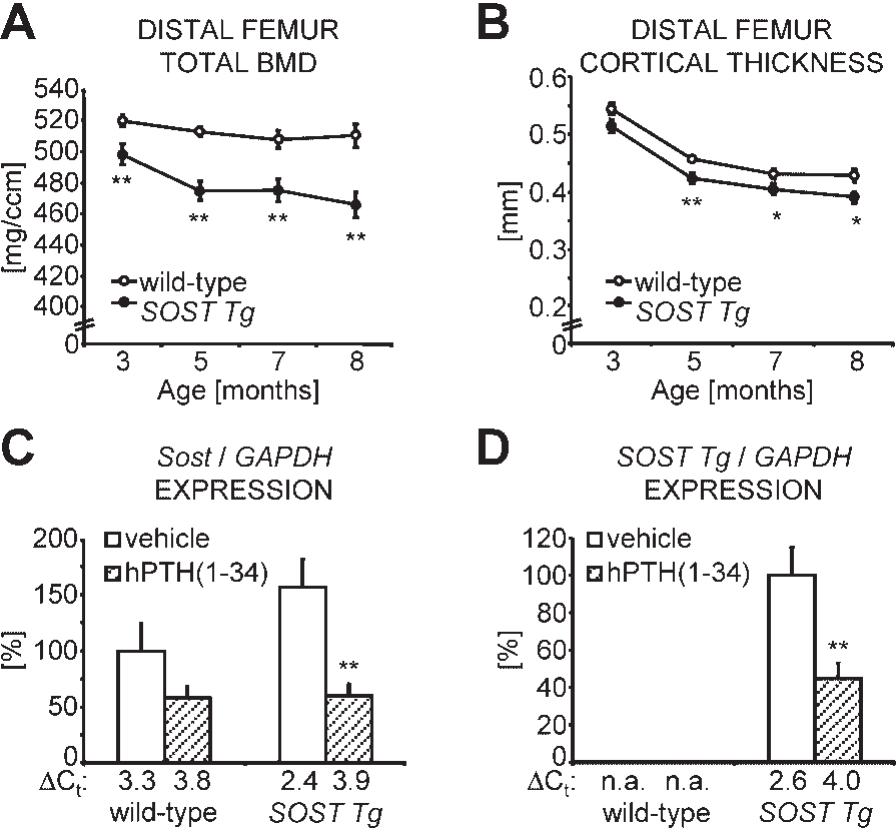
*SOST* overexpression results in osteopenia, and *SOST* is suppressed by PTH. Cross-sectional total BMD (*A*) and cortical thickness (*B*) in the distal femur metaphysis were evaluated by monthly in vivo pQCT measurements of skeletally mature to aging wild-type (*white data points*) and *SOST Tg* (*filled data points*) littermate male mice. Relative expression of endogenous mouse *Sost* (*C*) and transgenic human *SOST* (*D*) in cortical bone of the tibia diaphysis normalized to *GAPDH* with the corresponding ΔC_t_ values indicated below the bars was determined by qPCR 4 hours following the final PTH (*hatched bars*) or vehicle (*white bars*) injection after 2 months of intermittent treatment with 100 µg/kg hPTH(1–34). Data represent means ± SEM. n.a. = not applicable. *p < .05, **p < .01; unpaired Student's *t* tests for *SOST Tg* versus wild-type (*A*, *B*) or PTH- versus vehicle-treated mice of the same genotype (*C*, *D*).

Consistent with *SOST* encoding a bone formation inhibitor and previously reported findings,(20) *SOST Tg* male mice expressing wild-type human *SOST* from a bacterial artificial chromosome (BAC) transgene under control of the human genomic context develop osteopenia with increasing age (Fig. 1*A*, *B*). Cross-sectional total BMD in the distal femur metaphysis was significantly lower in *SOST Tg* relative to wild-type littermate males, as evaluated in vivo by pQCT analyses during late-stage skeletal growth (3 months of age) and during skeletal aging (see Fig. 1*A*). Likewise, femoral cross-sectional cortical thickness was significantly decreased in aging *SOST Tg* mice compared with wild-type littermates (see Fig. 1*B*).

We then subjected 6-month-old *SOST Tg* mice and their wild-type littermate males to 2 months of intermittent PTH treatment using daily injections of 100 µg/kg hPTH(1–34) peptide. 4 hours after the final hPTH(1–34) application, the mice were sacrificed, and endogenous and transgenic *Sost* expression was determined by qPCR. Similar to previous reports,(11,12) endogenous *Sost* expression was suppressed by about 50% relative to vehicle-treated animals in cortical bone of PTH-treated 8-month-old wild-type and *SOST Tg* littermate mice (see Fig. 1*C*). Moreover, human *SOST* expressed from the BAC transgene in *SOST Tg* mice was significantly suppressed 4 hours after the last hPTH(1–34) injection, comparable with endogenous mouse *Sost* (see Fig. 1*D*). The fairly mild osteopenia observed under baseline conditions in *SOST Tg* mice (see Fig. 1*A*, *B*) is in line with the moderate overexpression of *SOST* under control of the human *SOST* promoter and distant *cis*-regulatory elements present in the BAC transgene reaching expression levels within the physiologic range(28) (see Fig. 1*C*, *D*).

### Skeletal parameters of *SOST Tg* mice in response to PTH treatment

**Fig. 2:**
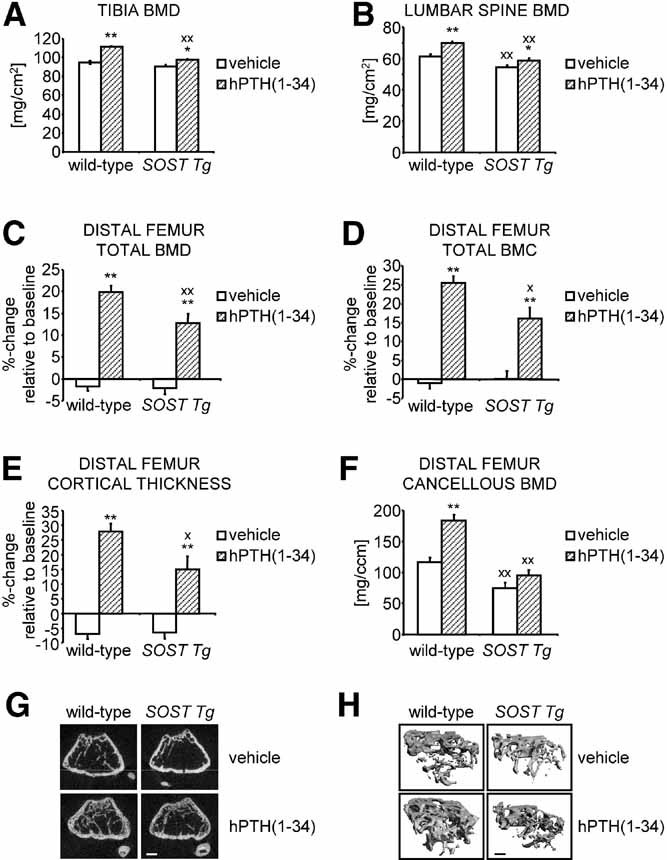
PTH-induced bone gain is blunted in *SOST Tg* mice. Following 2 months of high-dose intermittent PTH treatment, overall BMD in the tibia (*A*) and the lumbar vertebrae L1–4 (*B*) was determined in wild-type and *SOST Tg* mice by ex vivo DXA measurements. Percentage changes relative to baseline were calculated for cross-sectional total BMD (*C*), BMC (*D*), and cortical thickness (*E*) in the distal femur metaphysis, as measured by in vivo pQCT. Cancellous BMD in the distal femur metaphysis (*F*) was evaluated ex vivo by µCT, as illustrated by the 2D (*G*) and 3D µCT reconstruction images (*H*). Data represent means ± SEM for vehicle- (*white bars*) and PTH-treated (*hatched bars*) animals. Scale bars: (*G*) 0.5 mm; (*H*) 0.25 mm.*^,X^*p* < .05; **^,XX^*p* < .01; unpaired Student's *t* tests for PTH- versus vehicle-treated mice of the same genotype (*, **) or *SOST Tg* versus wild-type mice of the same treatment group (^X^, ^XX^).

We analyzed the bone anabolic responses in the tibia and lumbar vertebrae of *SOST Tg* and wild-type littermate mice relative to vehicle-treated animals using ex vivo whole-bone DXA measurements. Intermittent PTH treatment significantly increased total BMD in the tibia and lumbar spine of wild-type (+17% and +14%, respectively) and *SOST Tg* (+8% at both sites) mice. However, PTH-induced bone gain was significantly attenuated by about 50% in the appendicular and axial skeleton of *SOST Tg* compared with wild-type littermate mice (Fig. 2*A*, *B*).

To characterize PTH-induced bone gain, we determined the percent changes in cross-sectional total BMD and BMC in the distal femur metaphysis relative to baseline using pQCT analyses (see Fig. 2*C–E*). In wild-type mice, total BMD (see Fig. 2*C*) and total BMC (see Fig. 2*D*) significantly increased by 20% and 26%, respectively. In *SOST Tg* littermate mice, intermittent PTH treatment also induced significant increases in total BMD (+13%; see Fig. 2*C*) and total BMC (+16%; see Fig. 2*D*), but the bone anabolic responses clearly were blunted compared with the wild-type littermates.

We then analyzed the bone phenotypic changes in the cortical and cancellous bone compartment of the distal femur metaphysis by pQCT and high-resolution µCT. In wild-type mice, high-dose intermittent PTH treatment significantly increased cross-sectional cortical thickness by 28% relative to baseline (see Fig. 2*E*, *G*). Cancellous BMD was significantly augmented by 58% relative to vehicle-treated wild-type littermates (see Fig. 2*F*, *H*). In *SOST Tg* mice, cortical thickness was significantly elevated by 15% relative to baseline in response to intermittent PTH treatment (see Fig. 2*E*, *G*), whereas cancellous BMD increased merely in a nonsignificant manner by 27% compared with vehicle-treated transgenic littermates (see Fig. 2*F*, *H*). Cancellous bone volume was significantly increased by 73% in PTH-treated wild-type animals, whereas it was only nonsignificantly increased by 29% in PTH-treated *SOST Tg* mice (Table 1). Both trabecular number (+12%) and thickness (+11%) were significantly increased in PTH-treated wild-type controls, whereas increases were nonsignificant in *SOST Tg* mice (+3% and +7%, respectively; see Table 1). Thus relative PTH-induced bone anabolic responses were reduced by about 50% or more in both bone compartments in *SOST Tg* compared with wild-type littermate males

**Table 1:** Bone Structure and Histomorphometric Indices in the Cancellous Bone Compartment of the Distal Femur Metaphysis of Wild-Type and *Sost Tg* Mice After 2 Months of Intermittent PTH Treatment

	Wild-type		*SOST Tg*	
		
	Vehicle	hPTH(1–34)	Vehicle	hPTH(1–34)
BV/TV (%)	8.4 ± 0.5	14.6 ± 0.9**	5.2 ± 0.6^XX^	6.7 ± 0.7^XX^
Tb.N (1/mm)	3.7 ± 0.1	4.1 ± 0.1**	3.4 ± 0.1	3.5 ± 0.1^XX^
Tb.Th (µm)	51.7 ± 1.3	57.2 ± 2.0*	50.4 ± 1.5	54.1 ± 1.6
dLS/BS (%)	33.3 ± 3.8	64.0 ± 1.2**	41.6 ± 2.7	65.6 ± 2.0**
MS/BS (%)	47.6 ± 2.9	69.1 ± 1.2**	51.7 ± 2.4	70.6 ± 1.9**
MAR (µm/d)	0.41 ± 0.01	0.69 ± 0.02**	0.38 ± 0.02	0.59 ± 0.02**^XX^

Data represent means ± SEM. BS = bone surface; BV = bone volume; dLS = double-labeled surface; MAR = mineral apposition rate; MS = mineralizing surface; Tb.N = trabecular number; Tb.Th = trabecular thickness; TV = tissue volume.

* *p* < .05

** *p* < .01 for PTH- versus vehicle-treated mice of the same genotype using unpaired Student's *t* tests

*** *p* < .01 for *SOST Tg* and wild-type mice with identical treatment using unpaired Student's *t* tests.

### Bone formation and resorption parameters of *SOST Tg* mice in response to PTH treatment

**Fig. 3:**
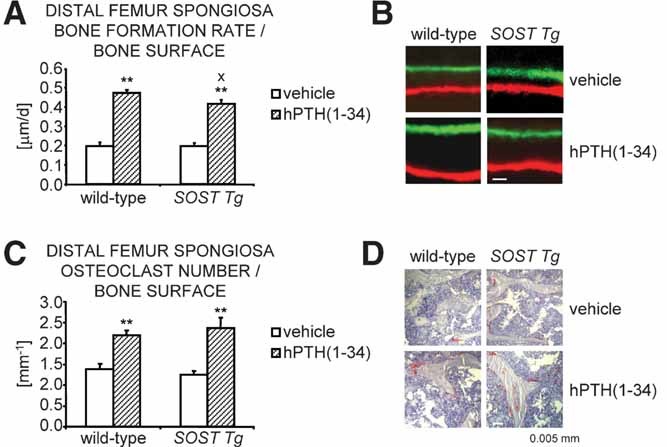
PTH-induced increase in bone formation rate is attenuated in *SOST Tg* mice. (*A*) Bone formation rates were determined by dynamic histomorphometry in the cancellous compartment of the distal femur metaphysis of vehicle- (*white bars*) and PTH-treated (*hatched bars*) wild-type and *SOST Tg* mice. (*B*) Representative images of fluorochrome-marker-labeled trabecular bone surfaces. (*C*) Osteoclast number was evaluated on TRAP-stained femoral sections, as illustrated by representative images of the cancellous compartment of the distal femur metaphysis (*D*). Scale bars: (*B*) 0.5 µm; (*D*) 5 µm. Data represent means ± SEM. ^X^*p* < .05; **p < 0.01; unpaired Student's *t* tests for PTH- versus vehicle-treated mice of the same genotype (**) or *SOST Tg* versus wild-type mice of the same treatment group (^X^).

We next analyzed bone formation parameters in the cancellous compartment of the distal femur metaphysis by dynamic bone histomorphometry based on double-fluorochrome-marker incorporation. In PTH-treated wild-type mice, mineralizing surface and mineral apposition rate were significantly elevated by 45% and 67% compared with vehicle-treated littermates, respectively (Fig. 3*B*; see also Table 1). Mineralizing surface (+36%) and mineral apposition rate (+54%) also were significantly greater in PTH-treated *SOST Tg* relative to vehicle-treated *SOST Tg* mice, but overall increases were smaller than in PTH-treated wild-type mice, and the mineral apposition rate was significantly lower in *SOST Tg* mice compared with wild-type mice (see Table 1 and Fig. 3*B*). Consequently, while in wild-type mice the bone formation rate was increased by 139% relative to vehicle-treated littermate mice, the PTH-induced increase was significantly less in *SOST Tg* mice (+111%; see Fig. 3*A*), further demonstrating that elevated *SOST* expression levels cause a reduction in the bone anabolic response to intermittent PTH treatment. In contrast, the PTH-dependent increase in osteoclast number was comparable between wild-type and *SOST Tg* mice, and no significant differences were observed in vehicle- or PTH-treated mice of either genotype (see Fig. 3*C*, *D*). Together these data demonstrate that overexpression of *SOST*, while being PTH-responsive, interferes with the full bone anabolic action of PTH in vivo owing to the higher net *Sost* expression levels present in *SOST Tg* mice following intermittent PTH treatment. To further corroborate this finding, we next assessed the skeletal responses to intermittent PTH treatment in homozygous *Sost* KO mice.

### Skeletal parameters of *Sost* KO mice

**Fig. 4:**
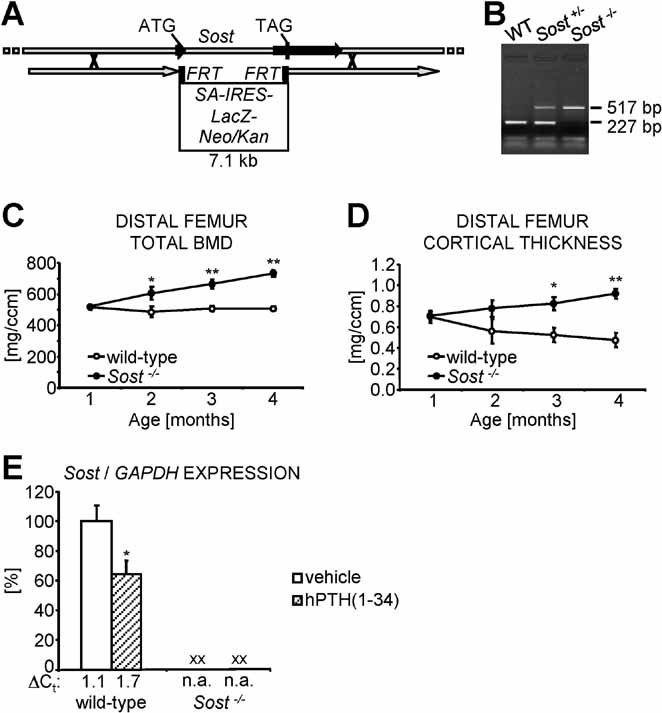
*Sost* loss-of-function results in a progressive high bone mass phenotype. (*A*) Genomic targeting scheme depicting the recombination strategy for generating the targeted *Sost* KO allele in which a 7.1-kb *LacZ-neomycin(Neo)/kanamycin(Kan) resistance* selection cassette replaces most of the *Sost* coding region, indicated by the ATG translation initiation and the TAG stop codon. Homology arms present in the targeting construct are represented by gray arrows; the 2 exons of the *Sost* gene are shown as black arrows. Flipase recognition target (FRT) sites for optional removal of the selection cassette are depicted as black boxes flanking the selection cassette. IRES = internal ribosome entry site; SA = splice acceptor site. (*B*) Genomic DNA was extracted from tail tips and used for genotyping of offspring obtained from heterozygous (*Sost*^+/−^) *Sost* KO breeding pairs to generate wild-type (WT), heterozygous, and homozygous (*Sost*^−/−^) *Sost* KO mice. Cross-sectional total BMD (*C*) and cortical thickness (*D*) in the distal femur metaphysis of wild-type (*white data points*) and *Sost*^−/−^ (*filled data points*) littermate male mice were evaluated at monthly intervals starting at 1 month of age until skeletal maturity. (*E*) 4 hours after the final PTH or vehicle application following 2 months of intermittent treatment with 100 µg/kg hPTH(1–34), the relative expression of *Sost* normalized to *GAPDH*, with the corresponding ΔC_t_ values indicated below the bars, was determined by qPCR in cortical bone of the femur diaphysis. Data represent means ± SEM. n.a. = not applicable. *p < .05, **p < .01; unpaired Student's *t* tests for *Sost*^−/−^ versus wild-type mice (*C*, *D*) or PTH- versus vehicle-treated mice of the same genotype (*E*). ^XX^*p* < .01; unpaired Student's *t* tests for *Sost*^−/−^ versus wild-type mice of the same treatment group (*E*).

*Sost* KO mice carry a targeted disruption of the 2-exon *Sost* gene in which the majority of the *Sost* coding region was replaced by an *IRES-LacZ-neomycin/kanamycin resistance* cassette (Fig. 4*A*). Heterozygous *Sost* KO (*Sost*^+/−^) mice were interbred with generate homozygous *Sost* KO (*Sost*^−/−^) and wild-type offspring (see Fig. 4*B*), and their respective bone phenotype was characterized by in vivo pQCT measurements starting at 1 month of age during skeletal growth to skeletal maturity at 4 months of age (see Fig. 4*C*, *D*). Consistent with *Sost* encoding a potent bone formation inhibitor, homozygous *Sost* KO mice develop a progressive high bone mass phenotype. From 2 months of age onward, *Sost*^−/−^ male mice displayed significantly higher cross-sectional total BMD (see Fig. 4*C*) and elevated cortical thickness (see Fig. 4*D*) in the distal femur metaphysis than their wild-type littermates.

To avoid a situation of saturated bone growth with bone filled marrow cavities possibly present in older *Sost*^−/−^ mice, we subjected 1.5-month-old *Sost*^−/−^ male mice and their wild-type littermates to 2 months of treatment with daily injections of 100 µg/kg hPTH(1–34). As expected, Sost expression was completely eliminated in homozygous *Sost* KO mice in both vehicle- and PTH-treated experimental groups, whereas wild-type littermate mice showed a significant reduction in Sost expression to about 60% of the level of vehicle-treated mice 4 hours after the final PTH application (see Fig. 4*E*).

### Skeletal parameters of *Sost* KO mice in response to PTH treatment

**Fig. 5:**
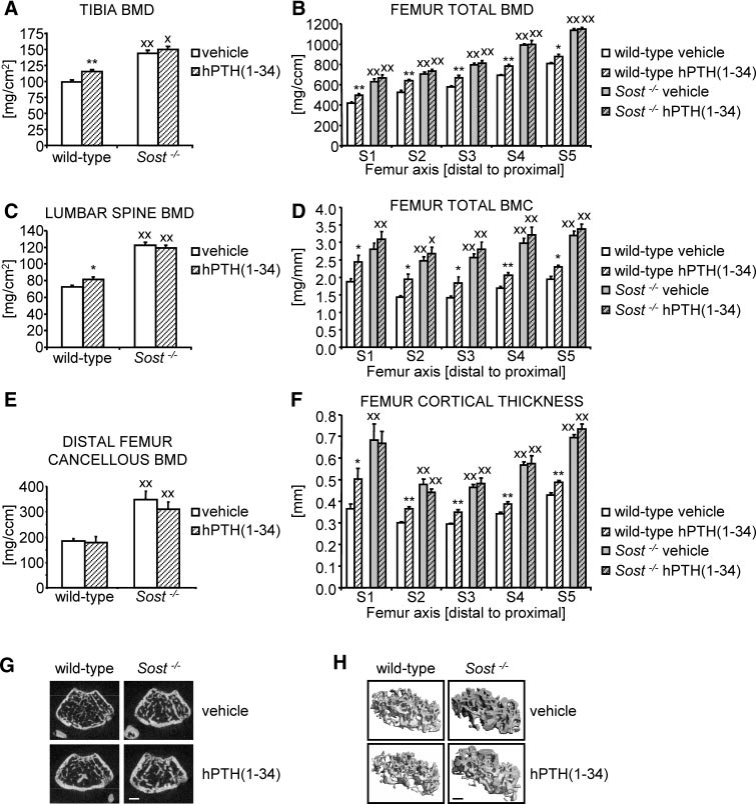
PTH-induced bone gain is blunted in *Sost*^−/−^ mice. Overall BMD in the tibia (*A*) and the lumbar vertebrae L1–4 (*C*) was determined in wild-type and *Sost* KO male mice ex vivo by DXA analyses following 2 months of high-dose intermittent PTH treatment. Femoral cross-sectional total BMD (*B*), BMC (*D*), and cortical thickness (*F*) were assessed by ex vivo pQCT measurements of five consecutive slices S1 to S5 distributed along the femoral axis at equal distances from the distal metaphysis (S1) to the proximal end of the diaphysis (S5). Cancellous BMD in the distal femur metaphysis (*E*) was evaluated ex vivo by µCT, as illustrated by the 2D (*G*) and 3D µCT reconstruction images (*H*). Scale bars: (*G*) 0.5 mm; (*H*) 0.25 mm. Data represent means ± SEM for vehicle- (*white bars*) and PTH-treated (*hatched bars*) animals. *^,X^*p* < .05; **^,XX^*p* < .01; unpaired Student's *t* tests for PTH- versus vehicle-treated mice of the same genotype (*, **) or *Sost*^−/−^ versus wild-type mice of the same treatment group (^X^, ^XX^).

We determined the bone phenotypic responses to intermittent PTH treatment using ex vivo whole-bone DXA analyses of the tibia and lumbar vertebrae. As observed previously in skeletally aging mice, 2 months of high-dose intermittent hPTH(1–34) treatment significantly increased total BMD in the appendicular and axial skeleton of skeletally growing wild-type mice by +16% in the tibia (Fig. 5*A*) and +13% in the lumbar spine (see Fig. 5*C*) compared with vehicle-treated littermates, respectively. Total BMD values of the tibia and lumbar vertebrae of vehicle- and PTH-treated *Sost*^−/−^ mice were significantly higher than those of correspondingly treated wild-type mice. However, PTH- and vehicle-treated *Sost*^−/−^ littermate mice did not differ significantly in their total BMD at either skeletal site, indicating a distinct blunting of PTH-induced bone anabolism in *Sost* KO mice.

To study the effects of high-dose PTH treatment in *Sost* KO mice in more detail, we next characterized the skeletal changes in the femur by ex vivo consecutive pQCT measurements along the femoral axis (see Fig. 5*B*, *D*, *F*). Femoral cross-sectional total BMD was significantly increased in PTH- relative to vehicle-treated wild-type mice, ranging from +20% in the distal metaphysis to about +10% at the proximal end of the femoral diaphysis (see Fig. 5*B*). Conversely, in *Sost*^−/−^ mice, intermittent PTH treatment did not result in a significant gain in total BMD at any femoral site analyzed relative to vehicle-treated littermate mice. Likewise, at all evaluated femoral sites, cross-sectional total BMC was significantly elevated in PTH- relative to vehicle-treated wild-type mice, whereas in *Sost*^−/−^ mice, PTH treatment induced only nonsignificant increases in total BMC compared with vehicle-treated littermate mice (see Fig. 5*D*). Thus bone anabolic responses to 2 months of high-dose PTH treatment are strongly attenuated in skeletally growing *Sost* KO mice.

To determine whether PTH-induced bone anabolism was equally blunted in both bone compartments of *Sost*^−/−^ mice, we next determined the skeletal responses in femoral cancellous BMD and cross-sectional cortical thickness using ex vivo high-resolution µCT and multiple-slice pQCT analyses, respectively. Cross-sectional cortical thickness was significantly increased by about 15% to 40% in PTH- compared with vehicle-treated wild-type mice depending on the respective position analyzed (see Fig. 5*F*). In contrast, in *Sost*^−/−^ mice, no significant gain in cross-sectional cortical thickness was detectable in PTH- relative to vehicle-treated mice irrespective of the evaluated femoral site (see Fig. 5*F*). Interestingly, we did not detect any net increase in femoral cancellous BMD in response to intermittent PTH treatment in either wild-type or *Sost* KO mice (see Fig. 5*E*, *G*, *H*), most likely owing to the young age of the mice used in this study in contrast to the aged wild-type and *SOST Tg* mice described earlier (see Fig. 2*F*). Trabecular number actually was significantly lower in either PTH-treated group (−16% in wild-type and −23% in *Sost*^−/−^ mice, respectively; Table 2). Trabecular thickness, however, was significantly increased in PTH-treated groups (+36% in wild-type and +31% in *Sost*^−/−^ mice, respectively), suggesting that increased bone formation occurred in response to PTH treatment. As a net outcome, cancellous bone volume was unchanged in PTH-treated wild-type animals and slightly decreased in PTH-treated *Sost*^−/−^ mice (−15%; see Table 2).

**Table 2:** Bone Structure and Histomorphometric Indices in the Cancellous Bone Compartment of the Distal Femur Metaphysis of Homozygous *Sost* KO and Wild-Type Mice After 2 Months of Intermittent PTH Treatment

	Wild-type		*Sost*^−/−^	
		
	Vehicle	hPTH(1–34)	Vehicle	hPTH(1–34)
BV/TV (%)	13.1 ± 1.0	13.0 ± 2.2	30.2 ± 3.7^XX^	25.7 ± 2.9^XX^
Tb.N (1/mm)	5.1 ± 0.1	4.3 ± 0.3*	5.3 ± 0.2	4.1 ± 0.1**
Tb.Th (µm)	44.6 ± 1.1	60.8 ± 2.4**	83.4 ± 1.1^XX^	109.6 ± 4.3**^XX^
dLS/BS (%)	28.4 ± 5.1	61.4 ± 2.2**	64.5 ± 3.0^XX^	63.5 ± 3.7
MS/BS (%)	44.6 ± 2.7	65.5 ± 1.9**	70.8 ± 2.0^XX^	68.2 ± 3.5
MAR (µm/d)	0.49 ± 0.02	0.83 ± 0.06**	0.79 ± 0.07^XX^	1.12 ± 0.12*

Data represent means ± SEM. BS = bone surface; BV = bone volume; dLS = double labeled surface; MAR = mineral apposition rate; MS = mineralizing surface; Tb.N = trabecular number; Tb.Th = trabecular thickness; TV = tissue volume.

* *p* < .05

** *p* < .01 for PTH- versus vehicle-treated mice of the same genotype using unpaired Student's *t* tests

XX *p* < .01 for *Sost*^−/−^ and wild-type mice with identical treatment using unpaired Student's *t* tests.

### Bone formation and resorption parameters of *Sost* KO mice in response to PTH treatment

**Fig. 6:**
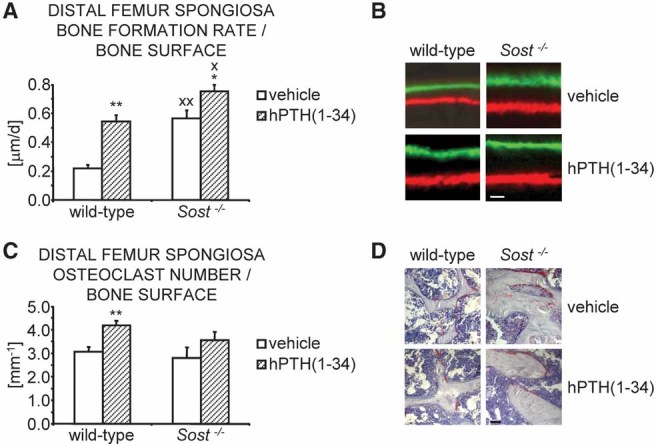
PTH-induced increase in bone formation rate is blunted in *Sost*^−/−^ mutant mice. (*A*) Bone formation rates were determined by dynamic histomorphometry in the cancellous compartment of the distal femur metaphysis of vehicle- (*white bars*) and PTH-treated (*hatched bars*) wild-type and *Sost*^−/−^ mice. (*B*) Representative images of fluorochrome-marker-labeled trabecular bone surfaces. (*C*) Osteoclast number was evaluated on TRAP-stained femoral sections, as illustrated by representative images of the cancellous compartment of the distal femur metaphysis (*D*). Scale bars: (*B*) 0.5 µm; (*D*) 5 µm. Data represent means ± SEM. *^,X^*p* < .05; **^,XX^*p* < .01; unpaired Student's *t* tests for PTH- versus vehicle-treated mice of the same genotype (*, **) or *Sost*^−/−^ versus wild-type mice of the same treatment group (^X^, ^XX^).

Using dynamic bone histomorphometry, we next analyzed boneformation parameters in the cancellous bone compartment of the distal femur metaphysis of *Sost*^−/−^ and wild-type littermate mice in response to 2 months of high-dose hPTH(1–34) treatment. Compared with wild-type mice, which showed a significant increase in mineralizing surface by almost 50% relative to vehicle-treated littermates, intermittent PTH treatment did not significantly alter the percentage of mineralizing surface in *Sost*^−/−^ mice relative to vehicle-treated *Sost*^−/−^ littermates (see Table 2). In contrast, intermittent PTH treatment caused a significant increase in mineral apposition rates in both wild-type and *Sost*^−/−^ mice relative to vehicle-treated control littermates (Fig. 6*B*; see also Table 2). However, PTH-dependent stimulation of mineral apposition rate increase was reduced by almost 40% in *Sost*^−/−^ mice (+42% relative to vehicle-treated KO mice) compared with wild-type littermates (+68% relative to vehicle-treated wild-type mice). As a consequence, bone formation rate increase was strongly blunted in *Sost*^−/−^ animals compared with wild-type littermate mice. While in wild-type mice intermittent PTH treatment augmented the bone formation rate to almost 150% of that of vehicle-treated wild-type mice, PTH treatment caused only a 33% increase in the bone formation rate of *Sost*^−/−^ mice relative to vehicle-treated *Sost* KO mice (see Fig. 6*A*). Together these data demonstrate that bone anabolic responses to intermittent PTH treatment are strongly reduced in homozygous *Sost* KO mice. As in aged mice, osteoclast number was significantly elevated in PTH- relative to vehicle-treated wild-type littermates (+37%; see Fig. 6*C*, *D*). In PTH-treated *Sost* KO mice, osteoclast number increased only nonsignificantly compared with vehicle-treated *Sost* KO mice (+28%), but no significant differences were detected between genotypes of the same treatment group (see Fig. 6*C*, *D*).

### Skeletal parameters of *Sost* KO mice in response to different anabolic doses of PTH

Finally, to exclude the possibility that the observed blunting of PTH-induced bone anabolism in *Sost*^−/−^ mice was merely a result of excessive stimulation of bone anabolic pathways triggered by the high daily dose of 100 µg/kg hPTH(1–34) and blunting would have not been detectable with lower doses of PTH, we repeated the 2 months of intermittent PTH treatment. We applied daily doses of 40 and 80 µg/kg of hPTH(1–34), respectively, 2 doses frequently used in mice to study PTH bone anabolic action in vivo. The lower dose of 40 µg/kg hPTH(1–34), while being relatively moderate for mice, is still known to induce bone anabolic responses in most mouse strains.

**Fig. 7:**
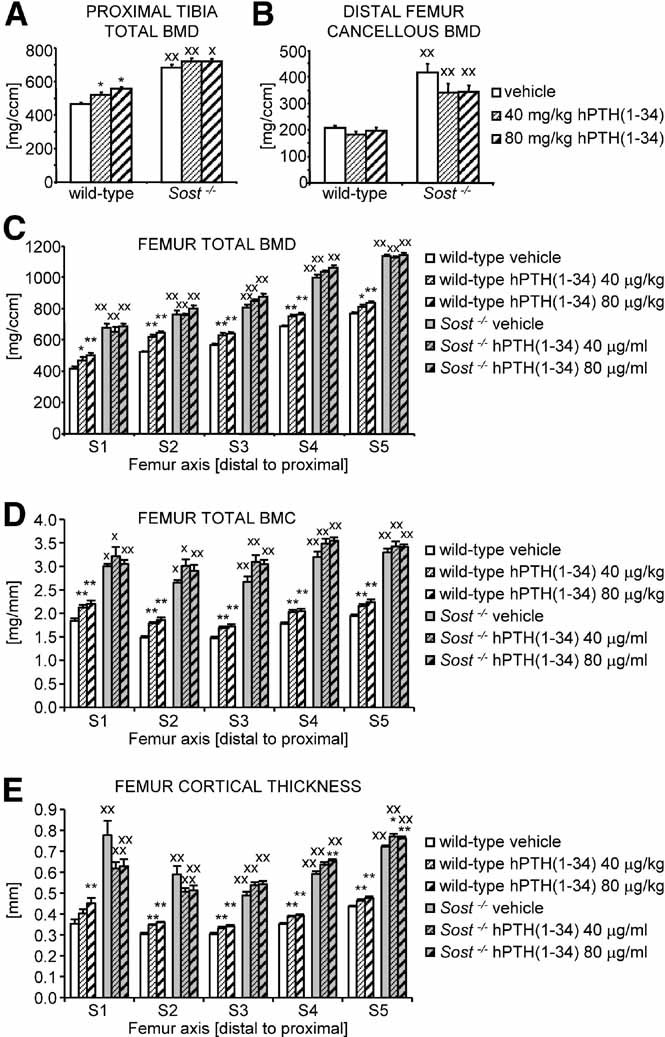
PTH-induced bone gain is blunted in *Sost*^−/−^ mice irrespective of the applied PTH dose. (*A*) Cross-sectional total BMD in the proximal tibia metaphysis was determined by in vivo pQCT analyses in wild-type and *Sost*^−/−^ male mice after 2 months of intermittent treatment with daily injections of vehicle (*white bars*) or 40 (*fine hatched bars*) or 80 µg/kg hPTH(1–34) (*bold hatched bars*). (*B*) Cancellous BMD was evaluated in the distal femur metaphysis by ex vivo µCT. (*C*) Femoral cross-sectional total BMD, total BMC (*D*), and cortical thickness (*E*) were evaluated by ex vivo pQCT analyses of five consecutive slices S1 to S5 distributed along the femoral axis at equal distances from the distal metaphysis (S1) to the proximal end of the diaphysis (S5). Data represent means ± SEM. *^,X^*p* < .05; **^,XX^*p* < .01; unpaired Student's *t* tests for PTH- versus vehicle-treated mice of the same genotype (*, **) or *Sost*^−/−^ versus wild-type mice of the same treatment group (^X^, ^XX^).

Similar to our previous observations with 100 µg/kg hPTH(1–34) daily dose, PTH-induced increase in cross-sectional total BMD was strongly blunted in *Sost*^−/−^ mice independent of the PTH dose and skeletal site analyzed. Cross-sectional total BMD in the proximal tibia metaphysis was only nonsignificantly increased by about 5% to 6% in PTH- relative to vehicle-treated *Sost*^−/−^ mice (Fig. 7*A*). In contrast, wild-type mice revealed a significant 12% and 19% increase in cross-sectional total BMD in responses to 2 months of intermittent treatment with daily doses of 40 or 80 µg/kg hPTH(1–34), respectively (see Fig. 7*A*). Furthermore, using multiple-slice pQCT measurements along the femoral axis, we found only small, nonsignificant changes in cross-sectional total BMD and BMC in *Sost*^−/−^ mice, whereas in wild-type mice treated with daily doses of 40 or 80 µg/kg hPTH(1–34), total BMD was elevated by 6% to 24% and total BMC by 11% to 26% relative to vehicle-treated littermates depending on the respective dose and femoral site analyzed (see Fig. 7*C*, *D*). As with 100 µg/kg hPTH(1–34) intermittent treatment, no net cancellous bone gain was noticeable in either genotype compared with vehicle-treated control mice (Table 3; see also Fig. 7*B*). Similar to the previous study, we observed mainly PTH-induced cortical bone gain by about 6% to 28% in wild-type mice depending on the respective dose and femoral site analyzed, which was blunted in *Sost*^−/−^ mice (see Fig. 7*E*). Consistent with the previous study, trabecular number was significantly lower in all PTH-treated groups (see Table 3). Trabecular thickness, however, was significantly increased in PTH-treated groups [+29% and 32% in wild-type mice and +13% and +23% in *Sost*^−/−^ mice treated with 40 or 80 µg/kg hPTH(1–34), respectively], suggesting that increased bone formation occurred in response to PTH treatment.

**Table 3:** Bone Structure Indices in the Cancellous Bone Compartment of the Distal Femur Metaphysis of Homozygous *Sost* KO and Wild-Type Mice After 2 Months of Intermittent PTH Treatment With Different PTH Doses

	Wild-type			*Sost*^−/−^		
		
	Vehicle	40 µg/kg hPTH(1–34)	80 µg/kg hPTH(1–34)	Vehicle	40 µg/kg hPTH(1–34)	80 µg/kg hPTH(1–34)
BV/TV (%)	14.2 ± 1.2	11.5 ± 0.9	12.9 ± 1.3	36.8 ± 3.5^XX^	28.1 ± 3.7^XX^	28.0 ± 3.1^XX^
Tb.N (1/mm)	5.2 ± 0.1	4.0 ± 0.1**	4.2 ± 0.1**	6.1 ± 0.3^X^	5.0 ± 0.5	4.4 ± 0.2**
Tb.Th (µm)	46.8 ± 1.4	60.2 ± 2.5**	61.9 ± 3.2**	84.3 ± 2.4^XX^	95.3 ± 3.5*^XX^	104.4 ± 1.9**^XX^

Data represent means ± SEM. BV = bone volume; Tb.N = trabecular number; Tb.Th = trabecular thickness; TV = tissue volume.

* *p* < .05

** *p* < .01 for PTH-versus vehicle-treated mice of the same genotype using unpaired Student's *t* tests

X *p* < .5

XX *p* < .01 for *Sost*^−/−^ and wild-type mice with identical treatment using unpaired Student's *t* tests.

## Discussion

Recently, we and others found that PTH suppresses transcription of the osteocyte-specific bone formation inhibitor *Sost*.(11,12) Moreover, PTH treatment results in a decrease in the number of osteocytes expressing sclerostin, the protein product of the *Sost* gene.(12,29) Here, we show that PTH-induced bone anabolism is attenuated in mice with altered *Sost* expression levels.

In aging *SOST Tg* mice, PTH-induced cortical and cancellous bone gain was attenuated in a comparable manner. These mice are expected to express higher net levels of sclerostin protein owing to the presence of the human *SOST* transgene. Therefore, the observed blunting of bone apposition indicates that for the full PTH bone anabolic response, *Sost* expression has to be reduced to a threshold below the level present in *SOST Tg* mice following PTH application. Accordingly, one might speculate that the absolute *Sost* expression level attained by PTH suppression is of more relevance than the relative difference in *Sost* expression levels induced by PTH signaling. At the molecular level, PTH application downregulated transgenic human *SOST* expression by about 50%, similar to endogenous mouse *Sost* expression, suggesting evolutionary conservation of PTH-induced suppression of *SOST*, an observation of putative importance concerning the mechanism of action of intermittent PTH treatment applied in the clinic. It is conceivable that PTH-induced suppression of *SOST* is mediated in part via the distant downstream bone enhancer also present in the *SOST* BAC transgene of *SOST Tg* mice. Consistently, we recently identified a conserved myocyte enhancer factor 2 (MEF2) response element in the distant *SOST* bone enhancer that is required for enhancer activity and is sufficient to mediate PTH-mediated suppression of *SOST* enhancer activity in vitro.(30) In this respect, it will be interesting to address in future studies the relevance of the distant *SOST* bone enhancer and the role of MEF2 transcription factors for PTH-induced bone anabolism in vivo.

While PTH-induced cancellous bone volume gain was clearly blunted in *SOST Tg* mice, similar to the observed blunting in the cortical bone compartment, PTH-dependent increase in cancellous bone formation rate was comparatively less strongly attenuated. This could be explained by partly altered kinetics of induction of PTH-dependent anabolism in wild-type versus *SOST Tg* mice and/or by the reduced cancellous bone template present in osteopenic *SOST Tg* mice at baseline. Along this line, it has been suggested that PTH treatment is less effective at skeletal sites with a lower amount of preexisting trabecular bone.(31) Moreover, we cannot exclude that *Sost* inhibits not only bone anabolic pathways acting downstream of PTH but also affects parallel bone anabolic pathways independent of PTH action. However, bone formation rates did not differ between vehicle-treated wild-type and *SOST Tg* mice, whereas they were attenuated in PTH-treated *SOST Tg* mice compared with treated wild-type mice. Hence it seems unlikely that the observed blunting of PTH-induced bone gain in *SOST Tg* mice was merely the result of an independent superimposed negative effect on parallel bone anabolic pathways. To further consolidate this interpretation, we analyzed PTH-induced bone anabolism in *Sost* KO mice lacking Sost expression constitutively. If bone anabolic pathways inhibited by *Sost* action differed from bone anabolic pathways activated by intermittent PTH treatment, we would have expected to observe additive effects of PTH-induced bone anabolism and *Sost* loss-of-function-dependent bone gain. Instead, we found that PTH-induced bone gain was severely impaired in the skeleton of growing homozygous *Sost* KO mice, providing further evidence that *Sost* suppression contributes to PTH-induced bone gain.

Morphologically, PTH bone anabolic action appears to rely in part on the reactivation of quiescent lining cells into bone-forming cells that contribute to PTH-induced increase in mineralizing bone surface.(7,32) In addition, PTH-induced expansion of mineralizing bone surface is thought to result from increases in osteoblast number via enhanced osteoblastic precursor proliferation and/or differentiation,(5,6,10) as well as reduced osteoblast apoptosis.(8,9) In wild-type as well as in *SOST Tg* mice, the relative mineralizing surface increased to 65% to 70% in response to 2 months of high-dose intermittent PTH treatment. This corresponds to the level of mineralizing bone surface present in vehicle-treated homozygous *Sost* KO mice, which did not further expand in PTH-treated *Sost* KO littermates, raising the issue of whether an inherent biologic limit exists in vivo that restricts the extent of active bone forming surface. Presently, we cannot fully exclude that the severe blunting of the PTH-induced increase in mineralizing surface observed in *Sost* KO mice is partly due to such a putative inherent saturation point reached in homozygous *Sost* KO animals. However, the observed blunting of osteoblastic mineral apposition rate in PTH-treated *SOST Tg* mice cannot be explained by such a hypothetical mechanism. Moreover, while being attenuated, intermittent PTH treatment still increased mineral apposition rate in *Sost* KO mice, indicating that bone formation had not reached a terminal saturation point in *Sost* KO mice. Importantly, PTH-induced bone gain also was strongly blunted in *Sost* KO mice treated with lower doses of hPTH(1–34), excluding that the observed reduction of PTH-induced bone gain in homozygous *Sost* KO mice was only due to excessive stimulation of bone anabolic pathways triggered by the high PTH dose. Finally, since *Sost* KO mice display an elevated matrix apposition rate and mineralizing bone surface, it appears that *Sost* inhibits osteoblastic bone matrix deposition as well as negatively regulates osteoblast recruitment and/or survival consistent with previous findings.(21,33,34) The present study suggests that both aspects of *Sost* action are of relevance for PTH-induced bone anabolism because either was affected by altered *Sost* gene dosage.

Unexpectedly, we did not observe any net increase in femoral cancellous BMD in growing wild-type and *Sost* KO mice subjected to intermittent PTH treatment in two independent studies. Nonetheless, trabecular thickness and cancellous bone formation rates increased in a manner comparable with the elevated cortical thickness and total BMD gain observed in wild-type mice at all skeletal sites. Thus it appears that intermittent PTH treatment stimulated boneformation responses in both bone compartments to a similar extent but failed to induce sustainable cancellous bone gain. Failure to achieve net cancellous bone gain was related to a decrease in trabecular number in PTH-treated animals of either genotype. Moreover, osteoclast number increased significantly in PTH-treated wild-type and nonsignificantly in PTH-treated *Sost* KO mice, indicating that the rarefied trabecular number was the consequence of PTH-stimulated enhanced bone resorption. Interestingly, while PTH-induced percentage increase in osteoclast number was similar or even slightly higher in aged wild-type and *SOST Tg* mice at the end of the treatment period, this did not interfere with overall cancellous bone gain in these animals. Thus it appears that net cancellous bone gain induced by intermittent PTH treatment is regulated by additional factors that might vary with age. Indeed, the number of bone resorbing osteoclasts was approximately doubled in young wild-type and *Sost* deficient animals compared with aged wild-type and *SOST Tg* mice irrespective of treatment. Another aspect that might contribute to these variations is the difference in genetic background. This notion is supported by the variability in skeletal responses to intermittent PTH treatment observed in different mouse strains.(31,35) Since net cancellous bone gain was undetectable in PTH-treated mice of either genotype and bone resorption parameters were comparable between genotypes of the same treatment group, *Sost* does not appear to have a major impact on PTH-induced bone resorption. Similarly, we did not detect any significant differences in osteoclast number comparing wild-type and *SOST Tg* mice or the relative PTH-triggered increases thereof, consistent with previous findings implicating *Sost* in the regulation of bone formation but not bone resorption.(20,21)

In contrast to the net bone anabolic action of intermittent PTH treatment, continuous PTH application is mostly bone catabolic owing to increased bone turnover, with elevated osteoclastic bone resorption exceeding the PTH-induced osteoblastic bone formation increase,(36,37) whereas osteocytic *Sost* expression is suppressed in both situations.(12) Recently, the role of PTH signaling in osteocytes was addressed in mice overexpressing a constitutively active PTH1R variant selectively in osteocytes, mimicking part of the effects triggered by continuous systemic PTH exposure in vivo.(13) These transgenic mice displayed a bone-overgrowth phenotype that was characterized by elevated bone turnover, decreased *Sost* expression, and increased Wnt signaling. In adult bone, *Sost-*encoded sclerostin is speculated to function as a secreted inhibitor of β-catenin-dependent canonical Wnt signaling by binding to Lrp5 and Lrp6 Wnt coreceptors, preventing their association with the Wnt-Frizzled receptor complex.(38,39) Given the importance of β-catenin-dependent canonical Wnt signaling for bone homeostasis,(40–43) the role of Wnt signaling in PTH-induced bone anabolism has been addressed previously in vivo.(44–46) Similar to the findings presented here for *Sost* KO mice, deletion of the Wnt signaling antagonist secreted frizzled-related protein 1 attenuates PTH-induced bone anabolism in vivo,(46) suggesting that activation of Wnt signaling contributes to PTH bone anabolic action. In addition, accumulating in vitro and in vivo evidence suggests a possible role for direct interaction of PTH1R and canonical Wnt signaling pathways.(47–50) Together, these findings point to potential mechanisms as to why PTH-induced suppression of *Sost* might be required for the full bone anabolic action of PTH in vivo.

In summary, we have shown in the present study that the in vivo bone anabolic action of intermittent PTH treatment is attenuated in *Sost* gain- and loss-of-function mouse models. Thus PTH-mediated suppression of the PTH downstream target *Sost* appears to contribute to PTH-induced bone gain in vivo.
